# Assessment in the primary care of the State of São Paulo, Brazil: incipient actions in sexual and reproductive health

**DOI:** 10.11606/S1518-8787.2017051006711

**Published:** 2017-08-03

**Authors:** Mariana Arantes Nasser, Maria Ines Battistella Nemes, Marta Campagnoni Andrade, Rogério Ruscitto do Prado, Elen Rose Lodeiro Castanheira

**Affiliations:** I Centro de Saúde Escola Professor Samuel Barnsley Pessoa. Faculdade de Medicina. Universidade de São Paulo. São Paulo, SP, Brasil; II Departamento de Medicina Preventiva. Faculdade de Medicina. Universidade de São Paulo. São Paulo, SP, Brasil; III Departamento de Saúde Coletiva. Faculdade de Ciências Médicas da Santa Casa de São Paulo. São Paulo, SP, Brasil; IV Departamento de Cardiologia. Hospital Israelita Albert Einstein. São Paulo, SP, Brasil; V Departamento de Saúde Pública. Faculdade de Medicina de Botucatu. Universidade Estadual Paulista. Botucatu, SP, Brasil

**Keywords:** Program Evaluation, Health Services Evaluation, Sexual and Reproductive Health, Sexually Transmitted Diseases, prevention & control, Acquired Immunodeficiency Syndrome, prevention & control, Primary Health Care, Unified Health System

## Abstract

**OBJECTIVE:**

The objective of this study is to assess performance in sexual and reproductive health of primary health care services of the Brazilian Unified Health System, in the State of São Paulo, Brazil.

**METHODS:**

An evaluative framework was built for sexual and reproductive health with the categorization of 99 indicators in three domains: sexual and reproductive health promotion (25), sexually transmitted infections/AIDS prevention and care (43), and reproductive health care (31). This framework was applied to assess the services responses to the questionnaire of Quality Evaluation of Primary Health Care in the Municipalities of São Paulo State (QualiAB), in 2010. Percentages were calculated for positive responses to indicators and performance in the sexual and reproductive health dimension, according to domains, and their contribution to the overall score in sexual and reproductive health (Friedman), relative participation (Dunn), and correlation (Spearman) was verified.

**RESULTS:**

Overall, 2,735 services participated in the study. They were located in 586 municipalities (distributed throughout the 17 regional health departments of São Paulo), of which 70.6% had fewer than 100,000 inhabitants. The overall average performance of these services for sexual and reproductive health is 56.8%. The actions are characterized by: prenatal with adequate beginning and exams, better organization for immediate rather than for late postnatal care, and selective reproductive planning for some contraceptives; prevention based on specific protection, limitations in the prevention of congenital syphilis, in the treatment of sexually transmitted infections, and in the screening of cervical and breast cancer; specific educational activities, with a restricted vulnerability approach, focus on sexuality over reproduction. The domain of reproductive health has greater participation in the overall score, followed by prevention/care and promotion. The three domains are correlated; the domain of prevention/care has the highest correlation with the other ones.

**CONCLUSIONS:**

The implementation of sexual and reproductive health in primary health care in the services studied is incipient. The revision of the purpose of the work, the dissemination of technologies, and the investing in permanent education are needed. The evaluative framework built can be used by the sexual and reproductive health program services and management in primary health care, thereby contributing to their actions.

## RESUMO

**OBJETIVO:**

Avaliar o desempenho em saúde sexual e reprodutiva de serviços de atenção primária à saúde do Sistema Único de Saúde, no estado de São Paulo.

**MÉTODOS:**

Construiu-se quadro avaliativo para a saúde sexual e reprodutiva com a categorização de 99 indicadores em três domínios: promoção à saúde sexual e reprodutiva (25), prevenção e assistência às doenças sexualmente transmissíveis/aids (43), e atenção à saúde reprodutiva (31). Esse quadro foi aplicado para avaliar as respostas dos serviços ao questionário Avaliação da Qualidade da Atenção Básica em Municípios de São Paulo (QualiAB), em 2010. Calcularam-se as porcentagens de respostas positivas aos indicadores e o desempenho na dimensão saúde sexual e reprodutiva, segundo os domínios; e verificou-se sua contribuição para o escore geral em saúde sexual e reprodutiva (Friedman), participação relativa (Dunn) e correlação (Spearman).

**RESULTADOS:**

Participaram 2.735 serviços, localizados em 586 municípios (distribuídos nos 17 departamentos regionais de saúde paulistas), dos quais 70,6% municípios com menos de 100.000 habitantes. A média geral do desempenho desses serviços para saúde sexual e reprodutiva é 56,8%. As ações são caracterizadas por: pré-natal com início e exames adequados, melhor organização para puerpério imediato do que tardio, e planejamento reprodutivo seletivo para alguns contraceptivos; prevenção baseada em proteção específica, limites na prevenção da sífilis congênita, no tratamento de doenças sexualmente transmissíveis, no rastreamento do câncer cervical e mamário; atividades educativas pontuais, com restrita abordagem das vulnerabilidades, predomínio do enfoque da sexualidade centrado na reprodução. O domínio saúde reprodutiva tem maior participação no escore geral, seguido de prevenção/assistência e promoção. Os três domínios estão correlacionados; o domínio prevenção/assistência apresenta as maiores correlações com os demais.

**CONCLUSÕES:**

A implementação da saúde sexual e reprodutiva na atenção primária à saúde nos serviços estudados é incipiente. É necessário rever finalidades do trabalho, disseminar tecnologias e investir em educação permanente. O quadro avaliativo construído pode ser utilizado pelos serviços e pela gestão do programa de saúde sexual e reprodutiva na atenção primária à saúde e contribuir para suas ações.

## INTRODUCTION

Primary health care (PHC) services are considered relevant to sexual and reproductive health (SRH) of individuals and population groups. The Program of Action of the IV International Conference on Population and Development^33^ and the Platform of Action of the IV World Conference on Women^32^, international milestones in the definition and visibility of SRH, point to PHC as a priority level. In Brazil, PHC is strategic to make SRH policies effective in the Brazilian Unified Health System (SUS)^12,22^.

The policy recommendations and delineation of the SRH programs are related to the attributes and purposes of the work in PHC^3,15,29^. Considered as “first and basic care”, PHC is characterized by its technological dimension^15^, the articulation of low-complexity material technologies and highly complex technical and organizational process technologies^29^, with the capacity to promote higher equality and efficiency to the health care system and a positive impact on the health of the population^30^. Oriented towards comprehensivenes^3^, it develops actions for health promotion, disease prevention, recovery, and rehabilitation. Because of its proximity to the territory – area of sanitary responsibility and geopolitical space^25^, headquarters of the life of subjects, with its needs and projects –, PHC is the preferable scenario of care practices^3,25,29,30^. Issues related to sexuality and reproduction trigger individual demands and requests from other sectors for PHC services; they have epidemiological relevance and are of collective interest for health education in the community^13,22^.

The constitution of the SRH field refers to expanded notions of health and sexual and reproductive rights, influenced by population and development policies, and the participation of social movements about sexuality and gender relations^7^. Reproductive health is considered the complete well-being of the reproductive functions and processes; its care includes methods, techniques, and services that contribute to the reproductive well-being and the prevention and resolution of problems. Sexual health aims to improve the quality of life and personal relations. It is not limited to reproductive counseling and assistance to persons with sexually transmitted infections (STI)^7^.

Brazil, a signatory of international conferences on rights in SRH^32,33^, has elaborated policies, programs, protocols, and recommendations related to the attributions of PHC to make SRH effective in the SUS, which guided this evaluative study^9^. Examples include the National Policies on STI and AIDS^16^, Comprehensive Care for Women’s Health^17^, Sexual Rights and Reproductive Rights^18^, Comprehensive Care for Men’s Health^21^, Primary Health Care^23^, Comprehensive Health of Lesbians, Gays, Bisexuals, Transvestites, and Transsexuals^24^, Legal Framework “Health, a Right of Adolescents”^20^, and the Notebooks of Primary Health Care 18, “HIV/AIDS, Hepatitis, and other STI”^19^ and 26, “Sexual and Reproductive Health”^22^.

In the State of São Paulo, PHC is characterized by the presence of an old, wide, heterogeneous network of services^5^; the central technical areas, such as the STI/AIDS State Program, are also traditional. Being a pioneer program in the country, it has integrated actions with PHC since 2000^26^.

Implementation of SRH care in PHC presents challenges such as: lack of discussion on this topic in health training^1^; difficulties in how professionals address this issue with users^13,22^; limited understanding of the SRH contents as basic health actions^22^; low integration between services^34^; focus on higher-risk groups over population strategies^34^; and little technological definition of prevention actions for STI and AIDS^12^, characterized by the individual values of professionals and conservatism^12^. In Brazil and in the State of São Paulo, proposals to overcome these challenges include publications, seminars, permanent education activities, and monitoring of the actions in PHC services^18,22,26^.

This article has the objective to assess the SRH performance of PHC services in the SUS in the State of São Paulo; it integrates the research Quality Evaluation of Primary Health Care in the Municipalities of São Paulo State (QualiAB), particularly, the Evaluation of SRH implementation in the PHC in the State of São Paulo^a^.

## METHODS

This study was developed as a health assessment with the creation of an evaluative framework for the SRH dimension in PHC. The answers to the questionnaire QualiAB, filled online by managers and services teams, in 2010, were used as source of data. The questionnaire was elaborated in evaluative research^5^, validated in 2007, and referenced by the health work theory – which considers health practices as technical and political intervention in reality^15^. The QualiAB is applicable to services of different organizational arrangements and it covers the various tasks of the PHC, enabling the assessment of specific themes^5^.

In 2010, the QualiAB was adopted as a tool to support the PHC management of the State Health Department (SES/SP)^5^. Adherence was open for all the municipal managers to register their services in the QualiAB system, and its dissemination predominantly focused on municipalities that had the Program of Primary Health Care Articulators, aimed at municipalities with less than 100,000 inhabitants^2^.

The evaluative framework for the SRH dimension consisted of the selection of answers to the questions of the QualiAB related to SRH, regarded as indicators and categorized according to the purposes of SRH promotion, STI/AIDS prevention and care, and reproductive health care (Table 1).

**Table 1:** Number of indicators for assessment of the sexual and reproductive health (SRH) dimension in primary health care (PHC), according to domains and subdomains.

Assessment framework for the SRH dimension in PHC			

Purpose of the actions in PHC	Domain	Subdomain	Indicator (n)
Well-being in the experience of sexuality and reproduction, with health education, individual empowerment, community participation and intersectoral activities. Guidance from the perspectives of human rights, vulnerability, sexual and reproductive rights, and gender.	SRH promotion	Health education and networking opportunities	8
		Investigation of situations of vulnerability and measures to overcome them	13
		Body, sexuality, and reproduction approach	4
	
Total indicators in the domain of SRH promotion			25
	
Prevention, diagnosis of STI/AIDS, care and support for individuals, considering the concept of vulnerability in their individual, social, and programmatic aspects, coordination with other services and sectors, importance of strategies for compliance, and contributions of health surveillance and information activities.	STI/AIDS prevention and care	STI/AIDS prevention and diagnosis	23
		Treatment, partnership with other services, and support to patients with STI/AIDS	11
		Health information and surveillance with opportunity for STI/AIDS prevention	9
	
Total indicators in the domain of STI/AIDS prevention and care			43
	
Health education and guarantee of reproductive choices, based on the perspectives of sexual and reproductive rights, and gender relations; prenatal and postnatal care for the family, women’s and children’s health, children, and families; reduction of morbidity and mortality; and humanization and attention to health care of reproductive and sexually related organs, including screening and diagnosis of neoplasms.	Reproductive health care	Reproductive planning	13
		Prenatal and postnatal care	12
		Health care of reproductive and sexually related organs	6
	
Total indicators in the domain of reproductive health care			31
	
Total indicators in the SRH dimension			99

Source: the authors, based on the study of Nasser MA (see footnote) and QualiAB 2010.

STI: Sexually Transmitted Infections; QualiAB: Questionnaire of Quality Evaluation of Primary Health Care in the Municipalities of São Paulo State

In order to characterize the practices of the services, the answers to the indicators generated a binary system (1 for what the service does; 0 for what it does not do). In each service, the answers to the indicators making up each subdomain were added; the total number of indicators making up the subdomain was the denominator for the value obtained, with the result varying between zero and 100. This procedure was repeated for each domain and for the SRH dimension, as a whole. Friedman test was used to compare the subdomains and domains in order to verify their contribution to the score created for the SRH dimension. A histogram was designed to show the distribution of services in the score for the SRH dimension. The relative participation of each domain or subdomain for the entire SRH dimension was subsequently obtained using Dunn’s nonparametric multiple comparisons for repeated measures. Spearman correlations were calculated to identify the existence of correlations between subdomains, domains, and the SRH dimension.

The QualiAB research, including this study, is in conformity with the standards of research ethics (CEP UNESP 435/2005; CEP FMUSP 471631/2013). Municipal managers agreed by term of compliance; the services participating signed the informed consent.

## RESULTS

From the 645 municipalities in the State of São Paulo, 586 (90.8%) were part of the QualiAB assessment, with the registration of 2,844 services, of which, 2,735 services (95%), distributed throughout the 17 Regional Health Departments of SES/SP, responded to the QualiAB^5^. According to population data of 2010, 55.6% of the participating services are located in municipalities with less than 50,000 inhabitants, 15.1% between 50,000 and 100,000, 12.7% between 100,000 and 200,000, 11.6% between 200,000 and 500,000, and 5.1%, with more than 500,000 inhabitants (IBGE, 2010)^5^. The coverage estimate achieved by the QualiAB is imprecise, because of consistencies in the existing records and their poor updating. Considering the number of 4,222 basic health units and 341 health centers, amounting to 4,563 basic services, registered in the National Register of Health Establishments in July 2010, the proportion of response to the QualiAB is 59.9% of the existing services.

Of the participating services, 56.6% are located in the urban periphery, 33.9% in the urban center, and 9.5% in the rural area. The distribution by organizational arrangement consists of 43.7% of Family Health Units (FHU), 32.0% of Basic Health Units (BHU), with or without specialties, 8.5% of BHU with Program of Community Health Agents (PCHA), 5.6% of BHU with family health team, 4.5% of BHU with specialties, 0.7% of upfront units, and 5.0% of other types.

The SRH actions developed in the PHC and the average performance of the group of services are presented in the domains of SRH promotion, STI/AIDS prevention and care, and reproductive health care (Table 2).

**Table 2:** Percentage of primary health care (PHC) services of the State of São Paulo, Brazil that report the fulfillment of sexual and reproductive health (SRH)* actions in 2010. (n = 2,735)

Subdomains	Indicators	%
Domain of sexual and reproductive health promotion		
Health education and networking opportunities	Annual thematic campaigns	82.2
	Educational activities with local institutions	58.5
	Educational activities with municipal departments	46.2
	Waiting room: discussions proposed by patients	9.8
	Health education for adolescents in schools	33.9
Investigation of situations of vulnerability and measures to overcome them	Educational activities on violence (C)	24.1
	Educational activities on alcohol and drugs (C)	35.5
	Care for the elderly includes guidelines on rights	38.5
	Diagnosis, guidance, and follow-up of cases of alcoholism (U)	10.5
	Diagnosis, guidance, and follow-up of women in situation of violence (U)	21.7
Body, sexuality, and reproduction approach	Guidance on development/changes in adolescence	38.8
	Care for the elderly includes approach to climacteric	52.0
	Care for the elderly includes approach to sexuality	36.1

Domain of STI/AIDS prevention and care		

STI/AIDS prevention and diagnosis	Application of vaccine for Hepatitis B (U)	74.5
	STI/AIDS prevention(C)	70.2
	STI/AIDS prevention(U)	80.3
	Adequacy of criteria for collecting Pap smear	5.4
	Opportunities for Pap smear collection	45.7
	Women’s health includes STI/AIDS prevention	86.4
	Education on sexuality and STI/AIDS prevention for adolescents	60.8
	STI/AIDS counseling for adults	64.8
	Two serologies for syphilis and HIV in prenatal care	68.7
	Distribution of condoms to the general population, according to demand	92.8
	Distribution of condoms includes female condoms	26.8
Treatment, partnership with other services, and support to patients with STI/AIDS	Application of penicillin – routine procedure	41.6
	Treatment of syphilis in pregnant women and their partners (U)	46.1
	Available drugs to treat STI	47.0
	In case of STI hypothesis: research, diagnosis, treatment (U)	67.4
	In case of STI hypothesis: syndromic treatment (U)	38.1
	In case of STI hypothesis: condom counseling (U)	83.7
	In case of STI hypothesis: serological testing counseling (U)	71.5
Health information and surveillance with opportunity for STI/AIDS prevention	Record of the number of vaccinations	73.6
	Record of the number of Pap smears	91.2
	Test results monitoring upon arrival (U)	49.9
	Nonexistence of congenital syphilis in prenatal (U), in 3 years	64.4

Domain of reproductive health care		

Reproductive planning	Family planning education (C)	55.4
	Family planning education (U)	72.5
	Oral contraceptive available in the last 6 months	83.4
	Injectable contraceptive available in the last 6 months	72.8
	Male condoms available in the last 6 months	94.9
	Female condoms available in the last 6 months	31.6
	Intrauterine device (IUD) available in the last 6 months	48.9
	Diaphragm available in the last 6 months	2.2
	Morning-after pill available in the last 6 months	33.9
	Referral for tubal ligation available in the last 6 months	71.8
	Referral for vasectomy available in the last 6 months	69.0
Prenatal and postnatal care	Pregnancy test (Pregnosticon or βHCG) is performed (U)	54.8
	Groups of pregnant adolescents (U)	28.8
	Most pregnant women begin prenatal in the 1st trimester of pregnancy	86.8
	Routine tests performed for all pregnant women	85.8
	Educational groups for pregnant women	61.3
	Time and place of immediate postpartum appointment scheduling	76.1
	Time and place of late postpartum appointment scheduling	63.0
Health care of reproductive and sexually related organs	Women’s health includes breast cancer prevention	91.7
	Physical examination for patients with a breast complaint	40.2
	Adequacy of criteria for mammogram referral	11.8
	Adult care includes prevention of prostate cancer	62.5

Source: The authors, based on the study of Nasser MA (see footnote) and QualiAB 2010.

C: activities conducted in the community; U: activities conducted in the unit; STI: sexually transmitted infections; QualiAB: Questionnaire of Quality Evaluation of Primary Health Care in the Municipalities of São Paulo State

* This table consists of a selection of actions relevant to this discussion. Complete data available in the study of Nasser MA (see footnote).

For SRH promotion, isolated activities are predominant in the actions of health education developed in the community and unit: campaigns restricted to the health service and sector, based on the transmission of information. Addressing the body and reproductive functions takes precedence over the discussion on rights and sexuality. Situations of vulnerability, such as alcohol abuse and violence, are more present in educational activities in the community than they are followed up in the units.

Regarding STI/AIDS prevention and care, specific protection actions, such as vaccination against hepatitis B and distribution of condoms to the general population, are among the main preventive measures, despite the scarce availability of female condoms. Educational activities for STI/AIDS prevention are more often in the units than in the community. Women are the main target audience, comparing adults and adolescents. There are limitations of screening for cervical-uterine cancer, because of the low criteria compliance and irregular offer of Pap smear. Syphilis and HIV diagnosis, during prenatal care, is compromised by improper collection of exams. Treatment of STI cases has limitations because of the insufficient adoption of syndromic treatment and the predominance of a diagnostic confirmation approach, requiring greater professional expertise and bringing risks to the adherence of the user. Use of condoms and serological testing are frequently advised. There are limitations in the availability of medications for STIs, the application of benzathine penicillin, and the treatment of pregnant women with syphilis and their partners. The nonexistence of cases of congenital syphilis is reported only in part of the units. Health surveillance activities, such as exam assessment, happen partially. The most frequently recorded information, such as vaccinations and collection of Pap smear, refers to productivity.

In reproductive health care, reproductive planning is a frequent activity in women’s health care, with a higher occurrence of health educational activities in the units than in the community. The availability of male condoms and oral contraceptives is high. The offer of surgical methods is frequent. On the other hand, female condoms and emergency contraception are scarcer. Most services offer prenatal as a scheduled activity, with admission of pregnant women in the first trimester and performance of the recommended tests. Health education actions for pregnant women are present in most units, some with groups for adolescents. Time and place of immediate postpartum appointment scheduling are usually more appropriate, than for late postpartum. The surveillance of no-show patients is more common for pregnant women when compared to puerperal women. Breast cancer approach often integrates the actions planned for women’s health; however, it rarely meets the criteria for mammogram referral. Men’s health actions include prevention of prostate cancer in many services.

**Figure 1:**
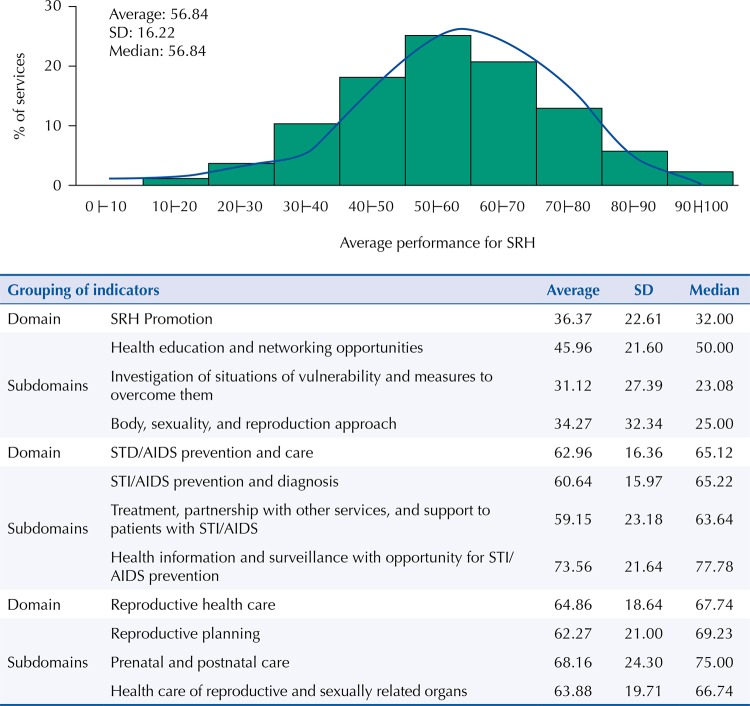
Performance of the primary health care (PHC) services in the sexual and reproductive health (SRH) dimensions: comparison between domains and subdomains and distribution of services according to the average performance in SRH (n = 2,735).

Source: the authors, based on the study of Nasser MA (see footnote) and QualiAB 2010.

STI: Sexually Transmitted Infections; QualiAB: Questionnaire of Quality Evaluation of Primary Health Care in the Municipalities of São Paulo State; SD: standard deviation.

The overall average performance of the services of PHC for the SRH dimension in São Paulo State is 56.8%; no service has an average higher than 91.9%. The distribution of services in the score for SRH originated a histogram that approaches the normal curve, which indicates the evaluation adequacy and allows the discrimination and description of the performance of the services using the arithmetic mean. Despite considerable variance, the analysis is significant because of the high number of indicators and participating services. Friedman test shows that reproductive health care is the domain with the highest contribution to the SRH dimension, followed by STI/AIDS prevention care and SRH promotion (Figure 1).

Friedman test: p < 0.001 for all values.

**Figure 2:**
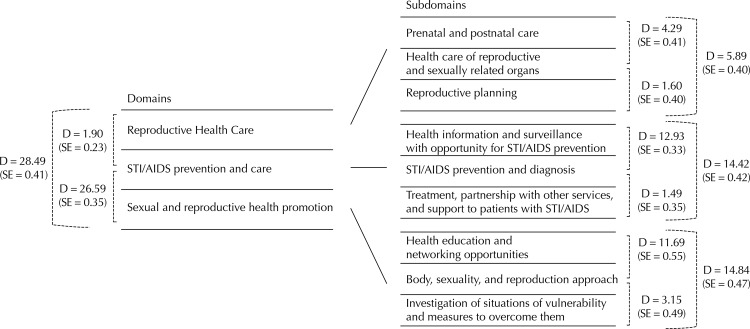
Average difference of the performance of primary health care (PHC) services between the domains of sexual and reproductive health (SRH) dimension and between the subdomains that compose each domain.

Source: the authors, based on the study of Nasser MA (see footnote) and QualiAB 2010.

STI: Sexually Transmitted Infections; QualiAB: Questionnaire of Quality Evaluation of Primary Health Care in the Municipalities of São Paulo State; D: average difference; SE: standard error

The emphasis of reproductive health care and the relative contribution of the other domains are confirmed by Dunn’s comparison, which shows the average difference of each domain of the SRH dimension in relation to the other ones. The comparison between the subdomains shows the distance between their participation in the composition of the domains. The average difference is attributed to the frequency of the actions performed (Figure 2).

Test: Dunn’s nonparametric multiple comparisons.

The representation of the average difference between each domain or subdomain is not to scale.

The correlation between all domains and subdomains comprising the SRH dimension is positive. The domain of STI/AIDS prevention and care is the only one with correlation greater than 0.6 with the other domains. The same happens with the subdomain of STI/AIDS prevention and diagnosis, which presents correlation greater than 0.5 with the other subdomains (except health care of reproductive and sexually related organs) (Table 3).

**Table 3:** Correlations between the sexual and reproductive health (SRH) dimension and each domain and subdomain to assess the SRH actions of primary health care (PHC) services in the State of São Paulo, Brazil. (n = 2,735)

Subdomains, domains, and SRH dimension	a	b	c	d	e	f	g	h	i	j	k	l	m
a. Health education and networking opportunities	1												
b. Investigation of situations of vulnerability and measures to overcome them	0.53	1											
c. Body, sexuality, and reproduction approach	0.48	0.63	1										
d. STI/AIDS prevention and diagnosis	*0.52*	*0.56*	*0.52*	*1*									
e. Treatment, partnership with other services, and support to patients with STI/AIDS	0.39	0.40	0.38	*0.55*	1								
f. Health information and surveillance with opportunity for STI/AIDS prevention	0.41	0.37	0.36	*0.51*	0.46	1							
g. Reproductive planning	0.32	0.32	0.33	*0.54*	0.50	0.38	1						
h. Prenatal and postnatal care	0.44	0.41	0.43	*0.54*	0.44	0.49	0.48	1					
i. Health care of reproductive and sexually related organs	0.29	0.38	0.35	*0.46*	0.43	0.35	0.37	0.39	1				
j. sexual and reproductive health Promotion	0.75	0.93	0.78	*0.64*	0.46	0.45	0.38	0.49	0.41	1			
k. STI/AIDS prevention and care	*0.54*	*0.56*	*0.53*	*0.88*	*0.81*	*0.73*	*0.58*	*0.59*	*0.51*	*0.64*	*1*		
l. Reproductive health care	0.44	0.45	0.46	*0.64*	0.57	0.50	0.84	0.83	0.59	0.53	*0.70*	1	
m. Sexual and reproductive health	0.66	0.75	0.68	*0.83*	0.71	0.65	0.66	0.72	0.57	0.84	*0.91*	0.83	1

Source: the authors, based on the study of Nasser MA (see footnote) and QualiAB 2010.

STI: Sexually Transmitted Infections; QualiAB: Questionnaire of Quality Evaluation of Primary Health Care in the Municipalities of São Paulo State

The domains (j, k, l) and the SRH dimension (m) were highlighted in bold to differentiate from the subdomains.

Values in italics correspond to higher correlations found for the domain of STI/AIDS prevention and care and for the subdomain of STI/AIDS prevention and diagnosis with the SRH dimension, other domains and subdomains.

Test: Spearman correlation. p < 0.001 for all values.

## DISCUSSION

Concerning the purposes of work in health care^3,29^, the greatest contribution of the domain of reproductive health care for the average performance in SRH reflects the role of tradition in the women’s health area in PHC. It reveals the power relations that characterize the care in this group^10,17^, notably regarding the protection of the maternal and child health and female body control^10,28^. The differences in the actions researched and the order of participation between the subdomains – prenatal and postnatal care, health care of reproductive and sexually related organs, and reproductive planning – possibly express an unequal recognition of these practices by both the health sector and the society. Actions seeking maternal and child mortality reduction and screening for neoplasms are regarded as more important than the ones seeking to guarantee reproductive choices in sexuality^8,10,28,31^. Training and know-how in technologies involved in each type of action are different: biomedical actions are more present than those of health communication and education actions^11,31^, with an appropriate offer of exams, compared to groups during prenatal care, for example. Actions commonly happen in the unit rather than in the community. There is a polarization between reproduction and sexuality^28^, as well as inadequacy in how sexual and reproductive rights and gender relations^10^are addressed indicated by the limitation in the supply of female condoms and emergency contraception.

The domain of STI/AIDS prevention and care occupies the second position in the SRH dimension. The achieved performance is related to PHC assignments in SRH and reflects challenges in its implementation: separation between reproduction and sexuality^28^, stigma related to STI/AIDS^8,27^, and difficulties to conduct care actions guided by the vulnerability and human rights framework^4,12,27^. A greater incorporation of measures for STI/AIDS prevention and diagnosis rather than for care expresses the historical demand for prevention actions in PHC^26^, as well as low recognition of STI care as its responsability^26^. Consequently, training and adoption of technological tools^14,15,26^ are necessary. The actions of health surveillance and information are a relatively high performance subdomain, with emphasis on data record related to production. However, these data require critical analysis, given the high frequency in the number of collection of Pap smear, in contrast with the low criteria compliance to prescribe the exam.

The lower number of actions of the domain of SRH promotion expresses both the challenge concerning the specific object and the practices of health promotion^3,4,31^, despite the fact they are PHC responsibility. The promotion of health requires work with social participation, approximation to the territory^25^, health education and communication methodologies^11,31^, interdisciplinarity, and intersectoriality^11^. Some particularities of SRH require understanding complex concepts – such as sexual and reproductive rights^7^, vulnerability^4^, and gender relations^7,10^ –, besides the need to adopt suitable technological tools^15,26^. There is also disarticulation between reproduction and sexuality^28^ and the predominance of informational over participatory strategies^11,31^. The difference between the educational activities about violence or alcohol and drug use and the follow-up of these cases shows the scarcity of tools for this operation^15^.

The positive correlation between the domains and subdomains of the SRH dimension shows that each one is related to the others, revealing the importance of comprehensiveness^3^ in the SRH care in PHC. The higher correlation of the domain of STI/AIDS prevention and care and, particularly, the subdomain of STI/AIDS prevention and diagnosis (in relation to the other domains and subdomains, respectively) show that both are good indicators for SRH. This characteristic can contribute to a better understanding of how they are implemented in the services. In addition, when taken as an investment and technological proposition focus, these indicators can foster improvements in SRH care practices in PHC. It can be assumed that work with STI/AIDS requires staff prepared to work with sensitive issues^8,27^ capable of addressing complex knowledge and techniques^14,15,26^. This can contribute to the handling of other objects in the health work, which require an approach that take into consideration user autonomy^10,11^, and situations of vulnerability^4,27^, as well as the establishment of interfaces between prescriptive measures and the projects of persons – combining technical attainment and practical success^3^.

The average performance of units of 56.8% for the SRH dimension exposes a discrepancy between what is accomplished by the services and what is proposed for PHC^22,32,33^ in order to achieve population impact^30^. The amount of assignments is a great challenge for PHC, and therefore reduction of expectations could be proposed. However, there are public policies and a SRH program for PHC in Brazil and in the State of São Paulo, which consider SRH actions essential and a responsibility of PHC^16-24^. Moreover, the very characteristics of PHC, including the articulation of health work purposes^3,29^ and proximity to the territory^25^, make SRH its object^13,22^.

The performance and integration of SRH actions, conducted by PHC services, are indicators of the implementation of the SRH program in PHC. Thus, the analysis of the results points to an incipient implementation. This is due to inadequate recognition of SRH as an object of PHC – not only in its extent, but above all, in the comprehensiveness of SRH –, and the need for review, improvement, and expansion of the incorporation of technologies to handle SRH in PHC practice.

Regarding the limitations of this study, the assessment focused predominantly on organizational components, which are necessary, yet not sufficient to appreciate quality. The use of previously collected data precluded the inclusion of some objects of the SRH dimension, such as men’s health actions and the issue of sexual diversity. The findings cannot be extended to all PHC services in São Paulo: the sample was not done randomly, but rather from the adherence of managers; municipalities with large population were under-represented; the city of São Paulo did not participate in the study. On the other hand, the total number of the participating services was high and there was a significant participation of small municipalities, reflecting both the political and administrative structure of São Paulo, as 81.2% (524) of municipalities have less than 50,000 inhabitants (IBGE, 2010) and the support of the Program of Primary Health Care Articulators^2^, addressed to small municipalities. The articulation with the SES/SP program, previously mentioned, facilitated the return of results and favored the responsiveness to the recommendations following it^5^.

Methodological possibilities were also present; with emphasis on the construction of a viable assessment, integrating a general questionnaire about PHC with easily understood explanations and recommendations, which may contribute to improving the organization of the work with SRH in PHC. Additionally, the study provided a review and update of the SRH dimension in the new 2016 version of the questionnaire QualiAB. The need to improve PHC in Brazil is well-known. The diversity of evaluative approaches – as represented by the tools QualiAB, *Autoavaliação para a Melhoria do Acesso e da Qualidade da Atenção Básica* (AMAQ), *Programa Nacional de Melhoria do Acesso e da Qualidade da Atenção Básica* (PMAQ), and Primary Care Assessment Tool (PCATool-Brasil) – contribute to this improvement^6^.

## CONCLUSIONS AND CONTRIBUTIONS FOR THE SRH PROGRAM IN PHC

Considering PHC as strategic for SRH is a challenge. This study makes possible the proposition of a theory for the SRH program in PHC, taking into account the action purposes, and presenting priority activities and useful tools for the work. The evaluative framework for SRH in PHC, which, from the assessment of empirical data, was a mediator in the construction of this theory, can be used as a tool, particularly for work planning or future assessments.

With this evaluation framework, the task of assessing practices displaying different characteristics regarding purpose, technology, definition, and tradition in PHC has been fulfilled. It resulted in the creation of an assessment presenting variability of indicators, robust domains and subdomains, and discrimination between services in what concerns performance in SRH actions. These attributions, as well as the insertion of the SRH dimension in a general questionnaire about PHC, make this a viable and replicable assessment. In addition, this assessment shows completeness and usefulness by a combined elaboration of explanations, judgment, and recommendations.

To affirm that the implementation of SRH actions in PHC of the São Paulo State is incipient – namely, beginning, and therefore still inadequate and insufficient – also means to bet on changes and improvements, which is a possibility inherent in everything that is new. The results and analysis of this assessment may contribute to this process both in the organization of the work in the services and in the management of SRH program in PHC. It is worth mentioning that this assessment allows for a thorough understanding and discussion of the care practices for SRH developed by PHC, the performance of the services, and also the awareness of the domain of STI/AIDS prevention and care as a core indicator for assessments of SRH and a possible focus of technological proposition. Recommendations for the SRH program in the SUS’s PHC of São Paulo State highlight the need to comprehend SRH in PHC, as well as review the work purposes, and by taking into account the distribution of the performance of the services, investments in training and design of tools should be considered.

## Footnotes

a Nasser MA. Avaliação da implementação de ações em saúde sexual e reprodutiva desenvolvidas em serviços de atenção primária à saúde no estado de São Paulo [these]. São Paulo (SP): Faculdade de Medicina da Universidade de São Paulo; 2015 [cited 2017 Mar 20]. Available from: http://www.teses.usp.br/teses/disponiveis/5/5137/tde-22012016-110316/pt-br.php
